# Intraoperative Discovery of Type II Choledochal Cyst, Partial Annular Pancreas, and Duodenal Stenosis in a 45‐Day‐Old Term Born Female Infant

**DOI:** 10.1155/crpe/5914582

**Published:** 2026-06-19

**Authors:** Andrea de Abreu e Gouvea, Michael Stellon, Teresa Chapman, Shelly M. Cook, Adam Brinkman

**Affiliations:** ^1^ University of Wisconsin School of Medicine and Public Health, Madison, Wisconsin, USA, uwm.edu; ^2^ Department of Surgery, University of Wisconsin School of Medicine and Public Health, Madison, Wisconsin, USA, uwm.edu; ^3^ Department of Radiology, University of Wisconsin School of Medicine and Public Health, Madison, Wisconsin, USA, uwm.edu; ^4^ Department of Pathology and Laboratory Medicine, University of Wisconsin School of Medicine and Public Health, Madison, Wisconsin, USA, uwm.edu

**Keywords:** annular pancreas, congenital biliary dilation, duodenal stenosis, pediatric surgery, Type II choledochal cysts, upper GI tract obstruction

## Abstract

Type II choledochal cysts (CCs), a rare form of congenital biliary dilation characterized as a true diverticulum along the extrahepatic duct, comprise less than 2% of all cases. The coexistence of CC, annular pancreas (AP), and duodenal stenosis (DS) has been scarcely reported. We present the case of a 45‐day‐old female with a rare combination of congenital upper gastrointestinal anomalies discovered intraoperatively after imaging identified malrotation with concern for possible volvulus. The patient underwent exploratory laparotomy revealing a Type II CC, partial AP, and DS. Management included cyst excision, diamond‐shaped duodenoduodenostomy, and Ladd procedure, after which the patient’s recovery was uneventful. This case highlights the importance of maintaining a broad differential in infants with upper gastrointestinal tract obstruction and aims to improve awareness of these associated congenital pathologies.

## 1. Introduction

Congenital anomalies of the gastrointestinal tract can present in isolation or in complex combinations that challenge clinical diagnosis and management [1]. Choledochal cysts (CCs), also referred to as congenital biliary cysts, result from abnormal development or dilation of the bile duct system with an estimated incidence of 1 in 100,000–150,000 in Western populations. Type II CCs, which are true diverticula of the common bile duct, comprise less than 2% of all cases [2]. Annular pancreas (AP) and duodenal stenosis (DS) are rare congenital foregut anomalies that typically present in infancy with signs of upper gastrointestinal tract obstruction. AP arises from malrotation of the ventral pancreatic bud and has a prevalence of approximately 1 in 15,000 [3]. DS is caused by incomplete recanalization of the duodenal lumen during 8–10th week of gestation and is a leading cause of neonatal intestinal obstruction occurring approximately 1 in 5000–10,000 live births [4, 5].

We report the case of a 45‐day‐old female who developed progressive feeding intolerance and bilious emesis six weeks after surgical repair of a congenital diaphragmatic hernia (CDH). Exploratory laparotomy revealed a Type II CC, partial AP, and DS. Although these anomalies share a common foregut embryologic origin and may co‐occur, the coexistence of these three distinct anomalies in a single patient is exceedingly rare, with few previously documented reports like it [6–8]. This case highlights the surgical considerations involved in managing multiple concurrent congenital foregut anomalies and the importance of thorough intraoperative evaluation.

## 2. Case Summary

A female infant, weighing 3.05 kg, was born at 40 weeks of gestation via spontaneous vaginal delivery at home to a G1P1 mother without prenatal care; the pregnancy was otherwise uncomplicated. She presented to a local emergency department shortly after birth in respiratory distress, requiring intubation and mechanical ventilation. A chest radiograph revealed a left CDH, and following stabilization, she was transferred to the neonatal intensive care unit (NICU) at a tertiary children’s hospital. A repeat chest radiograph confirmed the CDH, and an echocardiogram revealed a small secundum atrial septal defect, large ventricular septal defect, and bicuspid aortic valve. Given her cardiopulmonary status remained stable, surgical repair of the CDH was performed between 24 and 48 h after birth. Intraoperatively, a small (2.5 × 2.5 cm) diaphragmatic defect was identified in the left posterolateral position (Bochdalek) containing small bowel, colon, stomach, spleen, and a portion of the liver, all of which were reduced. At the time, the bowel was described as viable without areas of necrosis or pallor.

**FIGURE 1:**
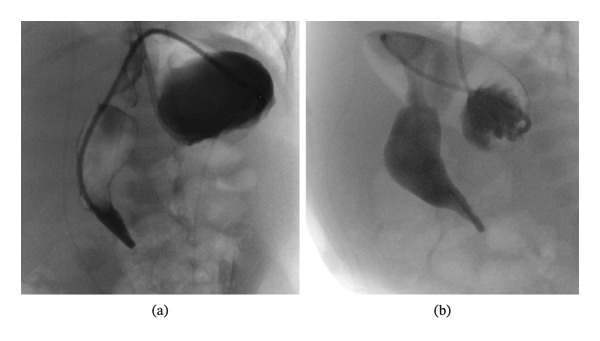
Nasojejunal enteric tube placement attempt under fluoroscopy confirms obstruction at the junction of the second and third portions of the duodenum. Fluoroscopic images of the upper abdomen in (a) supine AP and (b) right lateral decubitus positions with water‐soluble contrast injected through the tube delineate an abrupt tapering of the descending duodenum. Tubes could not be passed beyond this point of abrupt narrowing.

**FIGURE 2:**
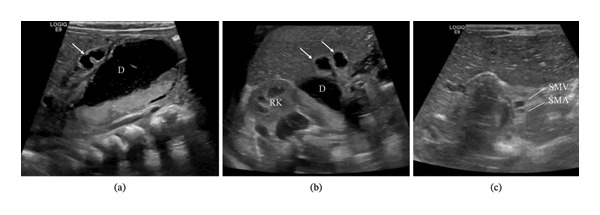
Limited abdominal ultrasound to assess for midgut volvulus. (a) Longitudinal grayscale image of the right upper quadrant near the gallbladder fossa shows a fluid‐distended viscous structure consistent with the dilated descending duodenum (D) containing layering intraluminal contents. Immediately adjacent to this was a tubular structure with anechoic contents (arrow) and wall folds, suggestive of a dilated cystic duct at the time of ultrasound interpretation. (b) Transverse grayscale image at the same level shows two parallel‐coursing tubular structures in the expected location of the gallbladder fossa (wide and narrow arrows), presumed to represent a small gallbladder and a distended cystic duct but later discovered to be a Type II choledochal cyst adjacent to the gallbladder at surgical exploration. RK = right kidney. (c) Transverse grayscale image of the upper mid abdomen slightly caudal to the above image shows an abnormal configuration of the superior mesenteric vein (SMV) immediately anterior to the superior mesenteric artery (SMA).

At 45 days of age, the patient developed feeding intolerance and increasing episodes of bilious emesis. Fluoroscopy during attempted nasojejunal (NJ) tube placement demonstrated duodenal dilation with failure of contrast to pass beyond the third portion of the duodenum, raising concern for intestinal obstruction, including the possibility of midgut volvulus in the setting of malrotation given the patient’s history of CDH (Figure 1). A focused abdominal ultrasound was performed to further assess for midgut volvulus, and the findings confirmed an intestinal rotational anomaly based on an abnormal relationship of the superior mesenteric artery and vein, but volvulus could not be definitively ruled out due to obscuration by bowel gas (Figure 2). The ultrasound was also notable for a tubular anechoic structure adjacent to the gallbladder, but its connection to the biliary tract could not be established, and its origin was unclear. Based on the folded wall pattern, the two parallel tubular structures in the gallbladder fossa were thought to represent a small gallbladder and dilated cystic duct. Given the etiologic consideration of midgut volvulus, the patient was taken emergently to the operating room for exploratory laparotomy with a plan for a possible Ladd procedure.

**FIGURE 3:**
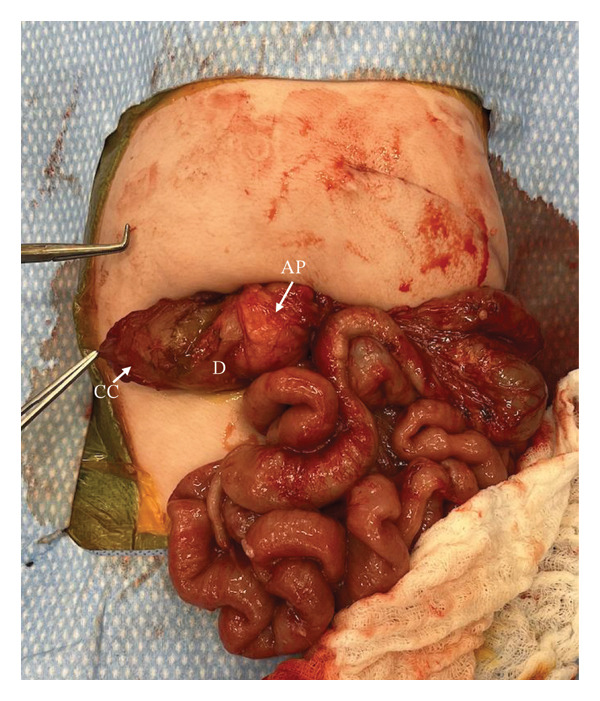
Intraoperative photograph obtained during exploratory laparotomy. A Type II choledochal cyst (CC) is visualized in the right upper quadrant, adjacent to the second portion of the duodenum (D). A partial annular pancreas (AP) is seen encircling the duodenum at the same level. While minimal duodenal narrowing was noted near these structures, the site of true obstruction was located in the third portion of the duodenum (not visible in this image), where duodenal stenosis was identified.

Intraoperatively, a transverse incision was made in the right upper quadrant, and the peritoneal cavity was entered. The small bowel was eviscerated, and approximately 25 min of lysis of adhesions was performed; the bowel appeared viable without evidence of necrosis or pallor. During exploration, a firm, egg‐sized, saccular structure was identified in the right upper quadrant densely adherent to the second portion of the duodenum and head of the pancreas, thought to be compressing the bowel. While initially suspected to be an enteric duplication cyst, further dissection revealed the mass to be a Type II CC measuring 5.3 × 2.4 × 1.5 cm. Upon decompression, bilious fluid was expressed. Once mobilized, the cyst was found to communicate with the common bile duct via a single abnormal duct, which was circumferentially dissected and ligated without narrowing of the extrahepatic biliary tree. The specimen was then sent to pathology for further evaluation. A partial AP encircling the duodenum was also identified, although not found to be the source of obstruction (Figure 3). DS was subsequently discovered in the third portion of the duodenum when a nasoenteric tube was unable to be advanced beyond the third portion of the duodenum in the operating room. This was subsequently addressed by performing a diamond‐shaped‐duodenoduodenostomy. Lastly, an intestinal rotational anomaly was confirmed, and a Ladd procedure was performed. Two months postoperatively, the patient was recovering well and tolerating enteric feeds at goals.

## 3. Discussion

Here, we present a case of upper gastrointestinal tract obstruction attributable to multiple congenital anomalies that required surgical exploration for both diagnosis and management. Preoperative diagnosis in this case was challenging. Initial imaging with fluoroscopic NJ tube placement attempt was diagnostic of an obstruction at the level of the mid duodenum, and subsequent abdominal ultrasound showed support for the presence of malrotation but was limited in identification of the precise cause of obstruction (Figures 1 and 2). Although ultrasound can be a feasible modality in evaluating for malrotation and midgut volvulus through the assessment of the SMA/SMV relationship and duodenal course in infants, it remains limited in detecting intrinsic lesions such as DS [9, 10]. In this case, the clinical concern for volvulus appropriately prompted urgent operative intervention, given the patient’s known CDH history, malrotation, and bilious emesis on exam. The limitations in current diagnostic pathways are highlighted by the fact that most intrinsic obstructions are often only confirmed during surgery.

On preoperative limited abdominal ultrasound of the right upper quadrant, two parallel tubular structures in the gallbladder fossa were assumed to be a small gallbladder and dilated cystic duct, based on a folded wall pattern. The possibility of a coincidental CC was not considered prior to surgery. Intraoperatively, a mass was identified in the right upper quadrant adherent to both the duodenum and the pancreas, requiring careful dissection and prolonging operative time to fully characterize the lesion and confirm that its mass effect was not the source of obstruction (Figure 3). The incidental discovery of the Type II CC necessitated intraoperative decision‐making regarding immediate excision versus deferred management; resection was performed given its biliary communication and anatomic complexity. A partial AP was also noted encircling the duodenum and was initially considered a potential cause of obstruction. However, the true source of obstruction was ultimately identified as DS in the third portion of the duodenum.

This underscores a key challenge in pediatric surgery, that even when multiple anatomic anomalies are present, only one may be functionally significant. Predicting which anomaly is clinically dominant is often not possible until direct visualization is achieved. Management of incidental discoveries during abdominal exploration must balance anatomic relationships and the risk of future complications—including obstruction, infection, or malignant transformation—against the additional operative time and morbidity associated with intervention. Moreover, applying a systematic intraoperative approach is critical to avoid overlooking additional diagnoses. This case demonstrates how unexpected intraoperative findings can significantly shift surgical priorities and complexity.

**FIGURE 4:**
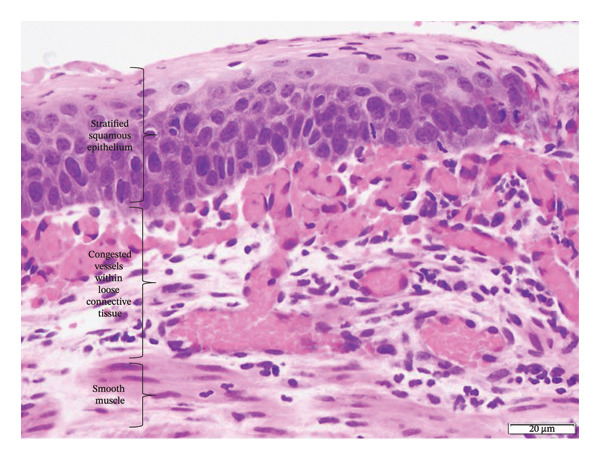
High power (400X) picture of the choledochal cyst wall showing stratified squamous epithelium overlying congested vessels within loose connective tissue, followed by smooth muscle. Although the cyst structure is similar to that of a gallbladder, its lining consists of squamous rather than columnar epithelium, the latter being more typically seen in the biliary system.

Histopathologic examination further contributed to the uniqueness of this case. The biliary system, including the gallbladder and biliary tree, is typically lined by a single layer of columnar epithelium, and prior reports indicate that CCs share this same epithelial morphology [11]. In contrast, this CC was lined by squamous epithelium, an atypical finding within the biliary tract (Figure 4). Such deviation from the expected columnar architecture is most often attributed to squamous metaplasia secondary to chronic inflammation, a process associated with an increased risk of malignant transformation [12]. In this case, however, there was no histologic evidence of chronic inflammation or transition zones, suggesting that the squamous lining may represent aberrant epithelial differentiation during foregut development, or possibly ectopic tissue.

Genetic testing provided additional context. Several weeks postoperatively, rapid whole‐exome sequencing identified a de novo pathogenic duplication at chromosome 14q24.3q32.33, along with a 14q32.33 deletion of uncertain significance. The coexistence of multiple foregut anomalies may reflect underlying disruptions in developmental pathways. Prior studies have linked duodenal anomalies to Trisomy 21 and other syndromes, and disruptions in the hedgehog signaling pathway, fibroblast growth factor, and retinoic acid signaling have been implicated in pancreatic and biliary malformations [13, 14]. Together, the genetic and histologic findings in this patient indicate broad disruptions in epithelial patterning and foregut morphogenesis contributing to the complex phenotype.

This case adds to the limited body of literature describing the coexistence of congenital foregut anomalies, particularly those involving the biliary tract, pancreas, and duodenum. Previously reported cases of CC associated with duodenal atresia have proposed shared embryologic mechanisms such as disrupted recanalization or aberrant epithelial proliferation of the foregut; however, those cases did not involve AP or malrotation, both of which further complicated the clinical and surgical landscape in our patient [2, 3]. Other reports have described a low–birth‐weight infant with AP, malrotation, and a Type I CC [4] but without intrinsic duodenal obstruction. Compared with these reports, our case is unique in the coexistence of a Type II CC, partial AP, DS, and malrotation in the setting of CDH. We recognize the spectrum of possible developmental disruptions and emphasize the importance of considering multiple coexisting anomalies when evaluating complex neonatal presentations.

## 4. Conclusion

This case reinforces the importance of maintaining a broad differential diagnosis and recognizing that congenital anomalies may copresent in complex, unpredictable ways. When multiple rare anomalies are present, a strong understanding of embryology and potential genetic associations can provide insight into their origin and aid in counseling and evaluation. Diagnostic imaging, while essential, may be insufficient in fully characterizing intrinsic versus extrinsic obstructions, particularly in neonates and infants. In such cases, clinical judgment and a low threshold for operative exploration remain vital.

## Funding

No funding was received for this manuscript.

## Disclosure

The authors have nothing to disclose.

## Ethics Statement

This case report was conducted in accordance with the Declaration of Helsinki and applicable HIPAA privacy regulations. In accordance with the University of Wisconsin‐Madison institutional policy, IRB review was not required as this case report does not meet the regulatory definition of human subject research.

## Consent

Written informed consent was obtained from the patient’s legal guardian for publication of this case report and any accompanying images.

## Conflicts of Interest

The authors declare no conflicts of interest.

## Data Availability

Data sharing is not applicable to this article as no datasets were generated or analyzed during the current study.
